# Biochemical failure-free survival of ^18^F-rhPSMA-7 and ^18^F-flotufolastat PET-guided salvage radiotherapy for patients with recurrent prostate cancer

**DOI:** 10.1038/s41598-024-83074-3

**Published:** 2025-01-17

**Authors:** Marco M. E. Vogel, Isabel Rauscher, Jürgen E. Gschwend, Türkay Hekimsoy, Nicola Gabler, Charlotte Olufs, Calogero D’Alessandria, Jan C. Peeken, Stephanie E. Combs, Matthias Eiber

**Affiliations:** 1 Department of Radiation Oncology, TUM University Hospital rechts der Isar, TUM School of Medicine and Health, Technical University of Munich (TUM), Munich, Germany; 2 Institute for Radiation Medicine (IRM), Helmholtz Zentrum München, Neuherberg, Germany; 3 Department of Nuclear Medicine, TUM University Hospital rechts der Isar, TUM School of Medicine and Health, Technical University of Munich (TUM), Munich, Germany; 4 Department of Urology, TUM University Hospital rechts der Isar, TUM School of Medicine and Health, Technical University of Munich (TUM), Munich, Germany; 5 Deutsches Konsortium für Translationale Krebsforschung (DKTK), Partner Site Munich, Munich, Germany; 6 BZKF (Bavarian Cancer Research Center), Munich, Germany; 7 Department of Radiation Oncology, TUM University Hospital rechts der Isar, TUM School of Medicine and Health, Technical University of Munich (TUM), Ismaninger Strasse 22, 81675 Munich, Germany

**Keywords:** Prostate cancer, PSMA-PET, ^18^F-rhPSMA-7, ^18^F-flotufolastat, Salvage radiotherapy, Prostate cancer, Prostate cancer

## Abstract

Prostate-specific membrane antigen (PSMA)-targeted positron emission tomography (PET) has improved localization of prostate cancer (PC) lesions in biochemical recurrence (BCR) for salvage radiotherapy (SRT). We conducted a retrospective review of patients undergoing ^18^F-rhPSMA-7 or ^18^F-flotufolastat (^18^F-rhPSMA-7.3)-PET-guided SRT compared with conventional-SRT (C-SRT) without PET. We evaluated biochemical failure-free survival (bFS) and overall rates of bFS in 110 evaluable patients with recurrent PC after radical prostatectomy who received SRT. 82 patients received ^18^F-rhPSMA-7/^18^F-flotufolastat-PET-guided SRT and 28 received C-SRT. Median bFS for patients with ^18^F-rhPSMA-7/^18^F-flotufolastat-PET-guided SRT was not reached while median bFS was 45.6 months for patients with C-SRT (*p* = 0.101). %bFS were 95% (52/55) *vs* 87% (20/23), 90% (27/30) *vs* 75% (15/20), 89% (16/18) *vs* 68% (13/19) and 100% (3/3) *vs* 57% (8/14) for PET-guided *vs* C-SRT at 12, 24, 36, and 48 months, respectively. Among patients treated in the prostate bed only, median bFS was not reached for PSMA-PET-guided SRT (n = 52) *vs* 55.1 months in the C-SRT group (n = 25; *p* = 0.063). %bFS was greater for PSMA-PET-guided SRT than C-SRT at all evaluated timepoints. ^18^F-rhPSMA-7/^18^F-flotufolastat-guided SRT yielded favorable disease outcomes. Although statistical significance was not reached, likely due to the limited sample size in this preliminary analysis, our data illustrate potential for ^18^F-flotufolastat-PET-guided SRT.

## Introduction

More than a third of patients with prostate cancer experience biochemical recurrence after radical prostatectomy^1^. In the past, such patients typically received salvage radiotherapy (SRT) to the prostate bed and/or pelvic lymph nodes based on clinical parameters. However, the advent of sensitive prostate-specific membrane antigen (PSMA)-targeted positron emission tomography (PET) imaging^2^ now allows improved localization of disease recurrence. It helps to inform management decisions, tailor targeted treatment, and potentially improve patient outcomes.

For SRT, PSMA-PET enables treatment of the macroscopic disease (local recurrence or pelvic lymph nodes) with higher doses than unguided external beam radiation to the elective prostate bed or lymph nodes. With modern intensity-modulated radiation therapy (IMRT), a simultaneous-integrated boost (SIB) is possible, without prolonging the total treatment time. We recently demonstrated that toxicity rates with PSMA-PET guided dose-escalated SRT with SIB were similar to conventional SRT (C-SRT) without SIB^3^.

^18^F-Flotufolastat (^18^F-rhPSMA-7.3) is a high affinity PSMA-targeting PET diagnostic radiopharmaceutical developed from a radiohybrid technology platform^4^. It is one of four diastereoisomers of the isomeric mixture ^18^F-rhPSMA-7^5^. Early clinical retrospective data demonstrate both ^18^F-rhPSMA-7 and ^18^F-flotufolastat to have high sensitivity for the detection of prostate cancer lesions^6–8^. However, in preclinical studies, ^18^F-flotufolastat showed superior clearance from the blood pool, liver, and kidneys, as well as high tumor accumulation, which led to its selection for further clinical development^5–9^. Lower average urinary excretion for ^18^F-flotufolastat than reported for other renally-cleared PSMA-targeting PET-radiopharmaceuticals was confirmed by clinical data suggesting potential for improved lesion detectability in the pelvis^10–12^. Based on two prospective multicenter phase III clinical trials, ^18^F-flotufolastat was recently approved by the United States Food and Drug Administration^13^ and has been included in the recent update to the NCCN Guidelines^14^.

Since PSMA-PET imaging is widely used by radiation oncologists as part of SRT planning and data on outcome after ^18^F-rhPSMA-7 and ^18^F-flotufolastat PET-guided SRT are scarce, we aim to specifically explore the impact of ^18^F-rhPSMA-7 or ^18^F-flotufolastat PET-guided SRT on disease outcomes. Therefore, we compare biochemical failure-free survival (bFS) and disease control rates in patients who underwent ^18^F-rhPSMA-7 or ^18^F-flotufolastat PET-guided SRT with those undergoing C-SRT without PET at our center.

## Methods

Data from 369 patients who were treated between November 2001 and November 2022 at the University Hospital of the Technical University of Munich (TUM) were retrospectively reviewed between 08/2023 and 09/2023. Patients with relapse after radical prostatectomy who received SRT with or without PSMA-PET guidance were included. Since PSMA-PET has been recommended by international guidelines only recently and cost coverage still remains challenging, in our institution patients can be treated with and without pre-RT PSMA-PET. All patients had a post-radical prostatectomy PSA nadir of ≤ 0.1 ng/mL, hence patients with PSA persistence after surgery were not part of this analysis.

Patients with PET-tracers other than ^18^F-rhPSMA-7 or ^18^F-flotufolastat (i.e. ^68^Ga-PSMA-11, ^18^F-PSMA-1007, ^11^C-choline) were excluded. To ensure comparability between the two groups, patients with distant metastases and patients with 3D RT were excluded.

Relapse after radical prostatectomy was defined as two consecutive rises of PSA and/or positive PSMA-PET/computed tomography (CT). Since PSMA PET-imaging is recommended at PSA levels of ≥ 0.2 ng/mL after radical prostatectomy^15^, we only analyzed patients in both groups who met this definition^16^ to ensure comparability. In addition, patients with negative PET were not analyzed as in those cases imaging did not guide SRT. Using this approach we finally focused on two different cohorts: ^18^F-rhPSMA-7 or ^18^F-flotufolastat PET-guided SRT versus conventional SRT (C-SRT, without PSMA-imaging before SRT).

**Fig. 1:**
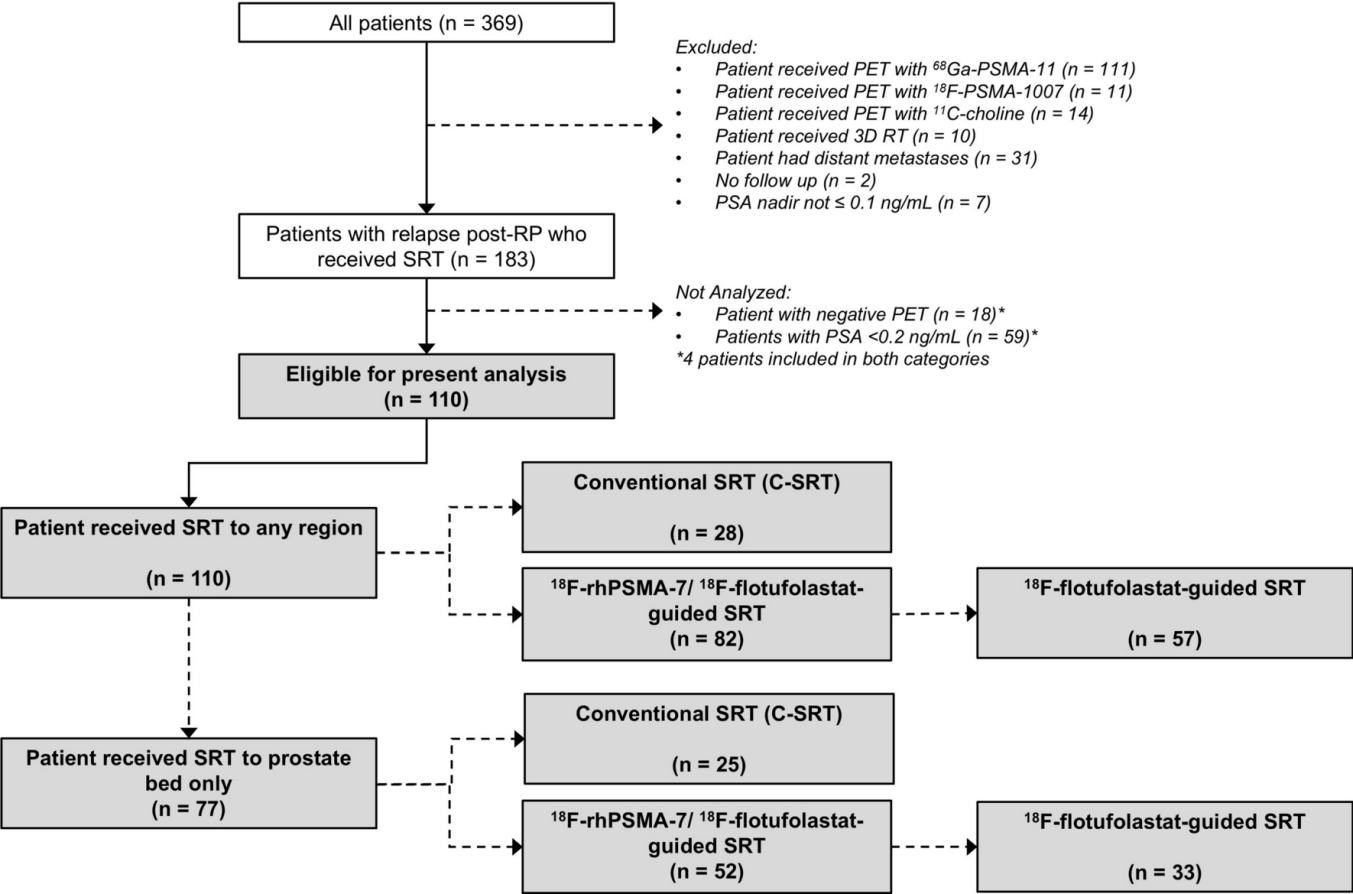
Patient flow diagram.

Patients were stratified according to the extent of the SRT (any region, or prostate bed only). Patient selection is presented as a flowchart in Fig. 1.

The institutional review board of the Technical University of Munich (TUM) approved this retrospective study (No. 564/19-S). We performed this analysis in compliance with the principles of the Declaration of Helsinki and its latter amendments. The need for informed consent was waived under the Bavarian Hospital Law (Bayerisches Krankenhausgesetz Art. 27 Abs. 4) due to the study’s retrospective and anonymous design. The administration of ^18^F-rhPSMA-7 and ^18^F-flotufolastat complied with national regulations and the responsible regulatory body (Government of Oberbayern).

### PSMA-PET

The patients underwent PET/CT imaging with either ^18^F-rhPSMA-7 or ^18^F-flotufolastat, performed according to the joint EANM and SNMMI guidelines^17^. Imaging was acquired in conjunction with either a diagnostic CT (n = 68) or magnetic resonance imaging (MRI) (n = 14).

All patients received a diagnostic CT scan after i.v. contrast injection (Iomeron 300, weight-adapted, 1.5 mL/Kg) and oral intake of diluted contrast medium (300 mg ioxitalamate [Telebrix; Guerbet]). Furosemide (20 mg i.v.) was administered to all patients at the time of tracer injection and patients were asked to void urine prior to the scan. PET scans were acquired in 3D mode with an acquisition time of 2 min per bed position in flow technique (equals 1.1 mm/s). Emission data were corrected for randoms, dead time, scatter, and attenuation and were reconstructed iteratively by an ordered-subsets expectation maximization algorithm (four iterations, eight subsets) followed by a post-reconstruction smoothing Gaussian filter (5 mm full width at one-half maximum).

All scans were interpreted by either a combination of a board-certified nuclear medicine physician and a board-certified radiologist or one dual-boarded nuclear medicine physician/radiologist. Focal tracer uptake higher than the surrounding background and not associated with physiologic uptake was interpreted as positive for prostate cancer lesions.

### Radiotherapy

Radiotherapy was performed with volumetric or helical IMRT and image-guided radiotherapy (IGRT) with daily online imaging. Planning CT and RT were performed following a protocol that aimed for a reproducible comfortably filled bladder and empty rectum.

A pelvic planning MRI scan was not performed as a standard procedure prior to initiation of radiotherapy.

Target delineation was conducted using the RTOG^18^ or EORTC^19^ guidelines. Planning target volumes (PTV) for the SIBs included a margin of 5–10 mm in addition to the gross tumor volume (GTV). Indication for additive androgen deprivation therapy (ADT) was discussed in a multidisciplinary tumor board and recommended thereafter to the patient.

### Treatment

We used the following dose concept^3^, when the dose constraints of the organs at risk allowed. The elective pelvic lymphatics were treated with 50.4 Gy in 1.8 Gy single doses (28 fractions). When patients showed lymph node metastases, we used a simultaneous integrated boost (SIB) to positive lymph nodes with 58.8 Gy in 2.1 Gy doses (28 fractions) or 61.6 Gy in 2.2 Gy doses (28 fractions) depending on proximity to organs at risk (e.g. bowels). The prostate bed was treated with 68 Gy in 2 Gy single doses (34 fractions). When a local recurrence was detected, it was irradiated with a SIB up to 76.5 Gy in 2.25 Gy single doses (34 fractions). However, changes to the total doses of the prostate bed, elective lymphatic pathways and lymph nodes were possible and at the discretion of the treating radiation oncologist.

### Statistical analyses

The primary endpoint was bFS, where biochemical failure was defined as a PSA progression (PSA nadir + 0.2 ng/mL with one confirmatory value). We also report overall rates of bFS (% bFS) at 12, 24, 36 and 48 months post-SRT.

To compare baseline characteristics in both groups we used a Pearson’s chi-square test or an independent-samples median test. Subgroup analyses were performed for the cohorts of patients who underwent ^18^F-rhPSMA-7 or ^18^F-flotufolastat PET. For the comparison of bFS, we used a Cox regression analysis adjusted for the use of additive ADT. For the analysis of median bFS, we used the Kaplan Meier method. The % bFS rates at 12, 24, 36 and 48 months were calculated using only data from patients for whom follow-up data were available at the respective time point. To compare doses with different fractionation schemes, we used the equivalent dose in 2 Gy fractions with an alpha/beta ratio of 1.5 Gy (EQD2 (1.5 Gy)). All statistical analyses were performed with SPSS version 21 (IBM, Armonk, USA). A p-value of < 0.05 was considered as statistically significant.

## Results

### Patients

In total, 110 of the 369 patients who were screened were evaluated in this analysis. Patients were treated between 09/2010 and 09/2021. Patient characteristics are presented in Table 1.

**Table 1:** Patient characteristics.

	All patients N = 110	Patients with C-SRT n = 28	Patients with ^18^F-rhPSMA-7/^18^F-flotufolastat-guided SRT n = 82	*p*
Age, years				
Median	72.0	69.5	73.0	0.126
Range	50–84	57–79	50–84	
Treatment Fields, n (%)				
PB	25 (23%)	25 (89%)	–	< 0.001*
PB + ePLN	3 (2.7%)	3 (11%)	–	
PB/SIB	52 (47%)	–	52 (63%)	
PB/SIB + ePLN	9 (8.2%)	–	9 (11%)	
PB + ePLN/SIB	7 (6.4%)	–	7 (8.5%)	
PB/SIB + ePLN/SIB	9 (8.2%)	–	9 (11%)	
ePLN/SIB	5 (4.5%)	–	5 (6.1%)	
Postoperative Tumor Classification, n (%)				
pT2a	9 (8.2%)	4 (14%)	5 (6.1%)	0.645
pT2b	4 (3.6%)	1 (3.6%)	3 (3.7%)	
pT2c	43 (39%)	8 (29%)	35 (43%)	
pT3	1 (0.9%)	0 (0.0%)	1 (1.2%)	
pT3a	30 (27%)	9 (32%)	21 (26%)	
pT3b	21 (19%)	6 (21%)	15 (18%)	
Missing	2 (1.8%)	0 (0.0%)	2 (2.4%)	
Postoperative Nodal Status, n (%)				
Negative (pN0)	95 (86%)	24 (86%)	71 (87%)	0.720
Positive (pN1)	11 (10%)	3 (11%)	8 (9.8%)	
Unknown (pNx)	2 (1.8%)	1 (3.6%)	1 (1.2%)	
Missing	2 (1.8%)	0 (0.0%)	2 (2.4%)	
Postoperative Surgical Margin, n (%)				
Negative (R0)	76 (69%)	17 (61%)	59 (72%)	0.446
Positive (R1)	23 (21%)	7 (25%)	16 (20%)	
Unknown (Rx)	6 (5.5%)	3 (11%)	3 (3.7%)	
Missing	5 (4.5%)	1 (3.6%)	4 (4.9%)	
ISUP Grade Group, n (%)				
1 (≤ 6)	10 (9.1%)	3 (11%)	7 (8.5%)	0.116
2 (3 + 4 = 7)	37 (34%)	9 (32%)	28 (34%)	
3 (4 + 3 = 7)	36 (33%)	5 (18%)	31 (38%)	
4 (8)	9 (8.2%)	3 (11%)	6 (7.3%)	
5 (9–10)	14 (13%)	7 (25%)	7 (8.5%)	
Missing	4 (3.6%)	1 (3.6%)	3 (3.7%)	
PSA before RT, ng/mL				
Median	0.39	0.32	0.45	0.029*
Range	0.20–22.00	0.20–3.97	0.20–22.00	
Additive ADT				
Yes	75 (68%)	23 (82%)	52 (63%)	0.066
No	35 (32%)	5 (18%)	30 (37%)	

* = significant result. PSA, prostate-specific antigen; RT, radiotherapy; ng/mL, nanogram/milliliter; ADT, androgen deprivation therapy; ePLN, elective pelvic lymph nodes; ISUP, International Society of Urological Pathology; PB, prostate bed; SIB, simultaneous-integrated boost.

### Imaging results, radiation doses and follow up

Of the 82 patients who underwent ^18^F-rhPSMA-7/^18^F-flotufolastat PET-guided SRT, local recurrence only, lymph node metastases only, and both local recurrence and lymph node metastases were present in 61/82 (74%), 12/82 (15%), and 9/82 (11%) patients, respectively.

Patients were treated with the median doses shown in Table 2 and were followed up for a median 22.6 months (range 1.0–139.0 months). Median follow up was significantly longer in the C-SRT cohort than in the ^18^F-rhPSMA-7/^18^F-flotufolastat-guided SRT cohort (22.2 months *vs* 47.3 months, respectively; *p* = 0.002).

**Table 2:** Radiation doses for patients with conventional salvage radiotherapy (C-SRT) and those with ^18^F-rhPSMA-7/^18^F-flotufolastat-guided salvage radiotherapy.

	Patients with C-SRT (n = 28)	Patients with ^18^F-rhPSMA-7/^18^F-flotufolastat-guided SRT (n = 82)
	Median (range) total dose in EQD2(α/β = 1.5 Gy), Gy	Median (range) total dose in EQD2(α/β = 1.5 Gy), Gy
Prostate Bed	68.0 (60.0–68.0)	68.0 (64.5–70.0)
Elective pelvic lymph nodes	47.5 (44.0–50.0)	47.5 (47.5–47.5)
PET-positive lymph nodes	–	60.5 (60.5–68.0)
PET-positive local recurrence	–	82.0 (68.0–82.0)

C-SRT, conventional salvage radiotherapy; EQD2, equivalent dose in 2 Gy fractions with an α/β ratio of 1.5 Gy; PET, positron emission tomography; SRT, salvage radiotherapy.

### Disease outcomes

#### All patients receiving SRT

**Fig. 2:**
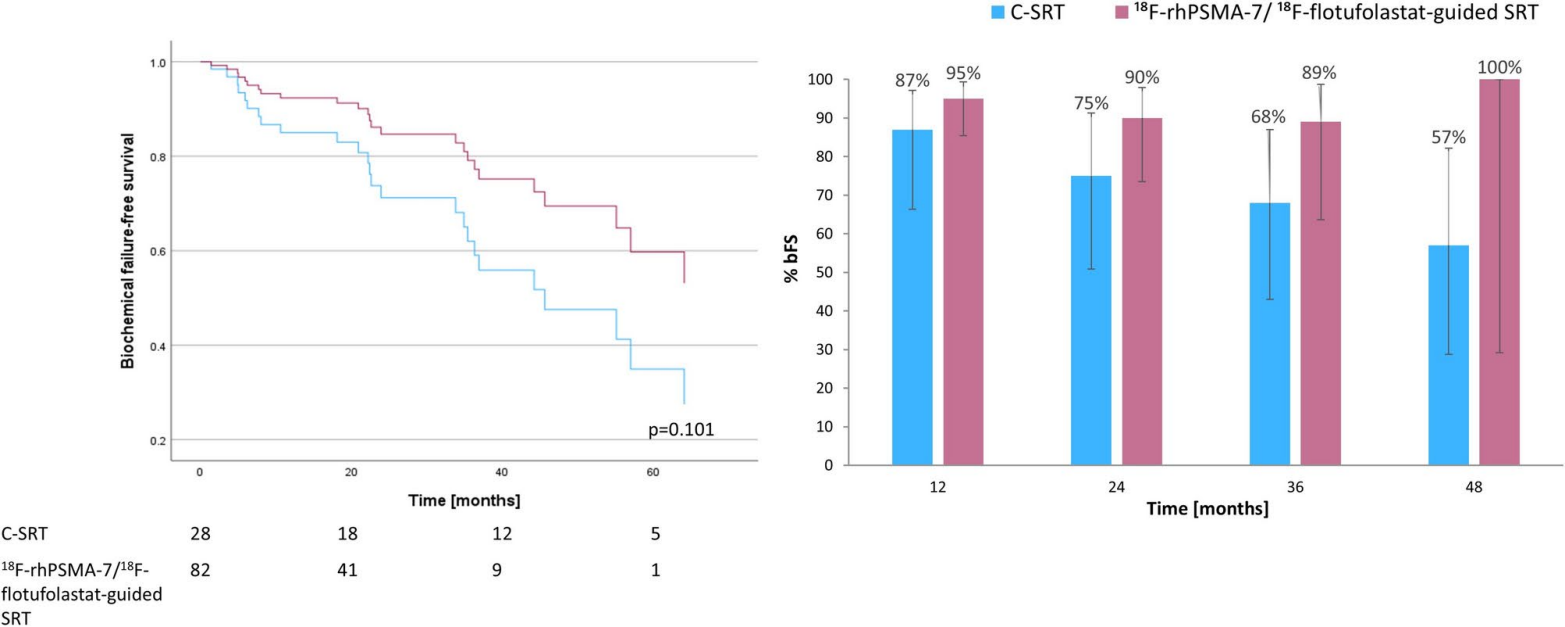
ADT-adjusted Cox regression of biochemical failure-free survival (left) and overall rates of biochemical failure-free survival at 12, 24, 36 and 48 months (right) for ^18^F-rhPSMA-7/^18^F-flotufolastat-guided salvage radiotherapy *vs* conventional salvage radiotherapy.

A Cox regression plot of bFS for ^18^F-rhPSMA-7/^18^F-flotufolastat-guided SRT (n = 82) *vs* C-SRT (n = 28) is presented in Fig. 2. The median bFS in the C-SRT cohort was 45.6 months (95% CI 27.51–63.69 months) and was not reached in the ^18^F-rhPSMA-7/^18^F-flotufolastat-guided SRT cohort (*p* = 0.101). The % bFS was 95% (52/55), 90% (27/30), 89% (16/18) and 100% (3/3), at 12, 24, 36 and 48 months, respectively among patients undergoing ^18^F-rhPSMA-7/^18^F-flotufolastat-guided SRT and 87% (20/23), 75% (15/20), 68% (13/19) and 57% (8/14), respectively in those receiving C-SRT (Fisher Exact test, *p* = 0.23–0.52).

**Fig. 3:**
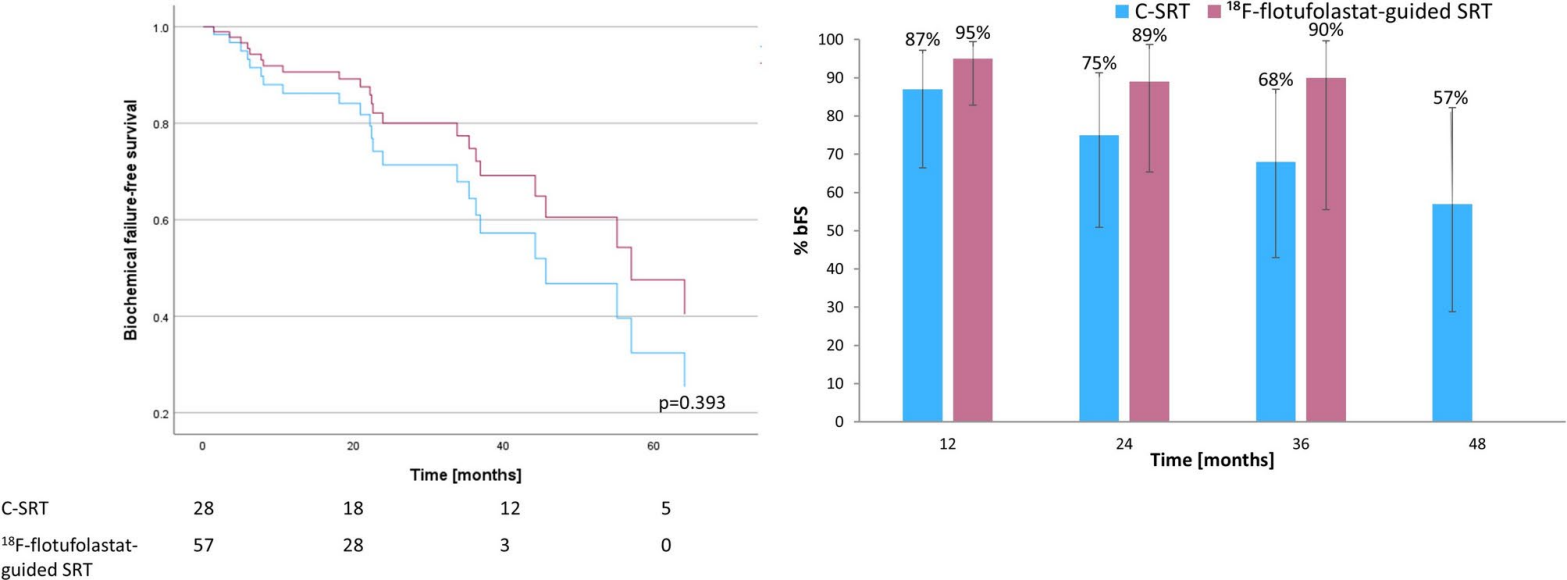
ADT-adjusted Cox regression of biochemical failure-free survival (left) and overall rates of biochemical failure-free survival at 12, 24, 36 and 48 months (right) for ^18^F-flotufolastat-guided salvage radiotherapy *vs* conventional salvage radiotherapy.

Figure 3 provides the comparison of bFS between C-SRT (n = 28) and the subset of patients who had ^18^F-flotufolastat-guided SRT (n = 57). The median bFS was 45.6 months (95% CI 27.51–63.69 months) in the C-SRT group and was not reached with the ^18^F-flotufolastat-guided SRT (*p* = 0.393). Overall rates of bFS (Fig. 3) were 95% (37/39), 89% (16/18) and 90% (9/10), at 12, 24, 36 months, respectively among patients undergoing ^18^F-flotufolastat-guided SRT (Fisher Exact test, *p* = 0.35–0.41 *vs* C-SRT). No patients were available for analysis at 48 months in the ^18^F-flotufolastat-guided SRT cohort.

#### Subgroup of patients receiving SRT to prostate bed only

**Fig. 4:**
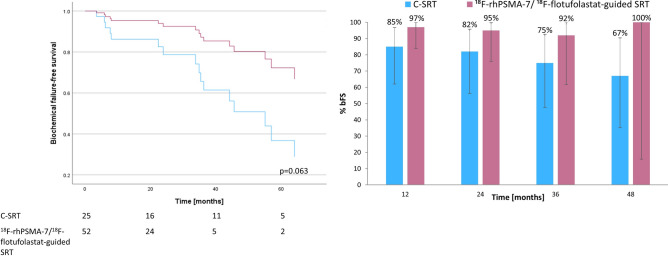
ADT-adjusted Cox regression of biochemical failure-free survival (left) and overall rates of biochemical failure-free survival at 12, 24, 36 and 48 months (right) for ^18^F-rhPSMA-7/^18^F-flotufolastat-guided salvage radiotherapy, *vs* conventional salvage radiotherapy in a subgroup of patients receiving SRT to the prostate bed only.

We further compared the subgroup of patients treated with ^18^F-rhPSMA-7/^18^F-flotufolastat-guided SRT (n = 52) versus C-SRT (n = 25) to the prostate bed only. Median bFS in the C-SRT cohort was 55.1 months (95% CI 40.90–69.30 months) and was not reached in the ^18^F-rhPSMA-7/^18^F-flotufolastat-guided SRT cohort (*p* = 0.063). A Cox regression plot is presented in Fig. 4.

The % bFS (Fig. 4) was 97% (31/32), 95% (20/21), 92% (11/12) and 100% (2/2), at 12, 24, 36 and 48 months, respectively among patients undergoing ^18^F-rhPSMA-7/^18^F-flotufolastat-guided SRT and 85% (17/20), 82% (14/17), 75% (12/16) and 67% (8/12), respectively in those receiving C-SRT (Fisher Exact test, *p* = 0.29–1.0).

**Fig. 5:**
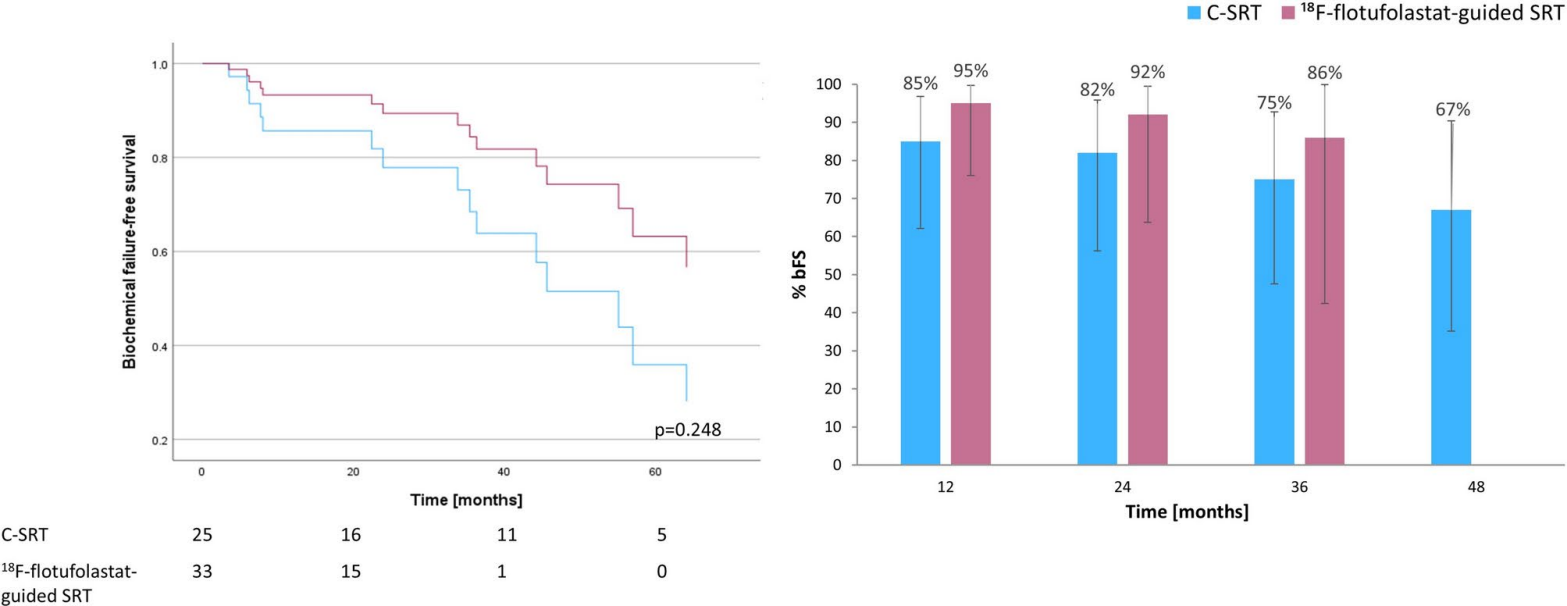
ADT-adjusted Cox regression of biochemical failure-free survival (left) and overall rates of biochemical failure-free survival at 12, 24, 36 and 48 months (right) for ^18^F-flotufolastat-guided salvage radiotherapy, *vs* conventional salvage radiotherapy in a subgroup of patients receiving SRT to the prostate bed only.

Figure 5 provides the comparison of bFS between C-SRT and the subset of patients who had ^18^F-flotufolastat-guided SRT in the subgroup of patients who received SRT to the prostate bed only. Median bFS was 55.1 months (95% CI 40.90–69.30 months) in the C-SRT (n = 25) and was not reached in the ^18^F-flotufolastat-guided SRT cohort (n = 33; *p* = 0.248). The % bFS (Fig. 5) was 95% (20/21), 92% (12/13) and 86% (6/7), at 12, 24, and 36 months, respectively among patients undergoing ^18^F-flotufolastat-guided SRT (Fisher Exact test, *p* = 0.34–0.41 *vs* C-SRT). No patients were available for analysis at 48 months in the ^18^F-flotufolastat-guided SRT cohort.

## Discussion

This retrospective analysis compared disease outcomes from ^18^F-rhPSMA-7/^18^F-flotufolastat-guided SRT with those of C-SRT in patients with biochemical recurrence of prostate cancer after radical prostatectomy. Our results show a trend towards favorable outcome in patients who underwent ^18^F-rhPSMA-7/^18^F-flotufolastat-guided SRT.

With the approval of three PSMA-PET pharmaceuticals in recent years^13,20,21^, PSMA-PET has become the mainstay for diagnostic imaging in patients with prostate cancer, particularly in the recurrence setting, and is recommended by national and international guidelines^14,16,22^. As prostate cancer dose–response data suggest the alpha/beta ratio for prostate cancer to be low^23^, target lesions are more resistant to low doses of SRT. Consequently, higher total doses to macroscopic lesions (local recurrence and/or pelvic lymph nodes) are increasingly being used for the treatment of patients with recurrent prostate cancer^24^. This has been facilitated by the accurate identification of target lesions for such dose escalation which was not possible prior to the advent of sensitive imaging modalities such as PSMA-PET.

**Fig. 6:**
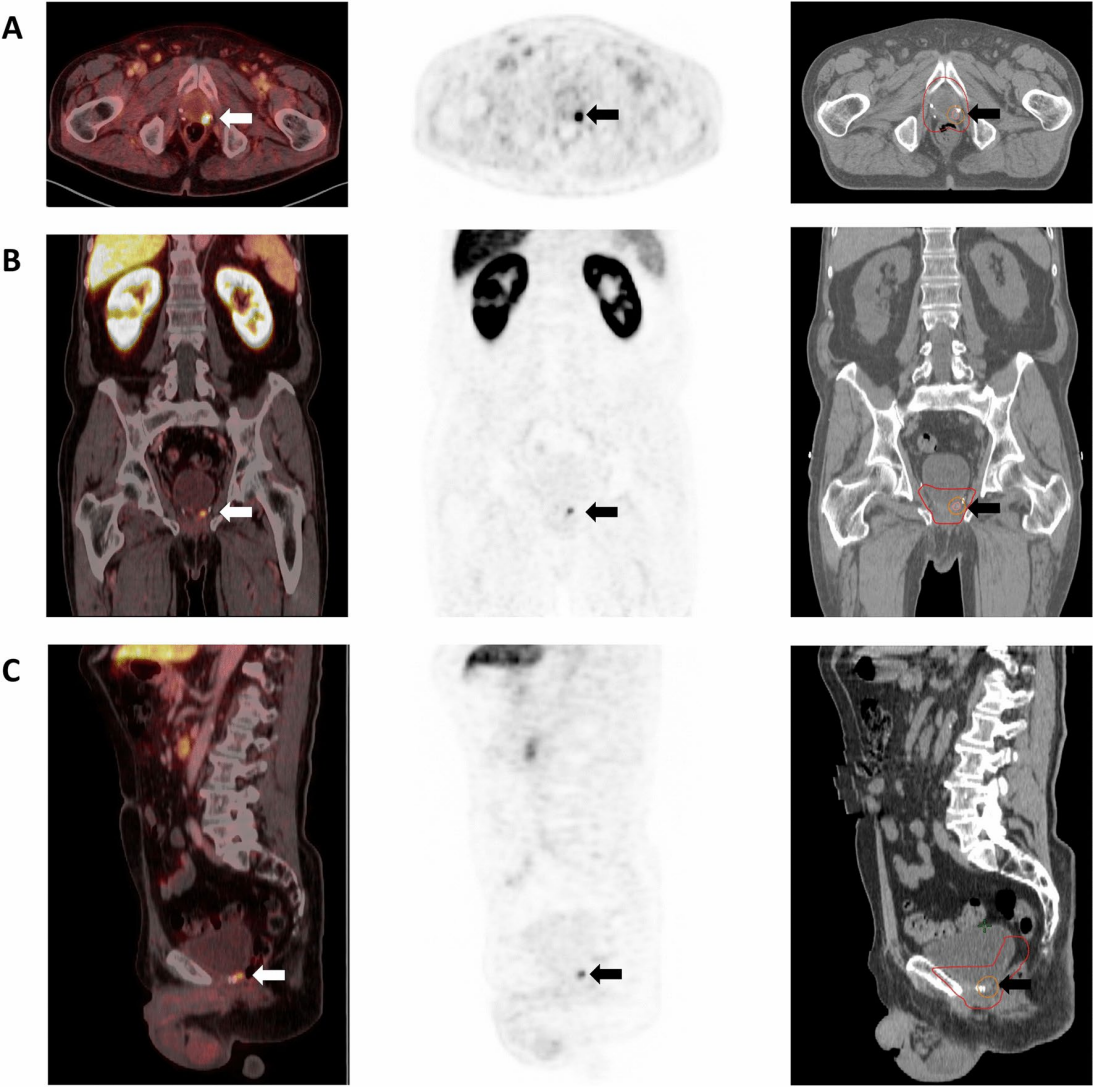
Example of a patient with ^18^F-flotufolastat-guided salvage radiotherapy for local recurrence located caudally to the bladder. The Figure shows an axial (**A**), coronal (**B**) and sagittal (**C**) plane of the PET/CT-fusion, PET sequence, and the resulting radiotherapy contouring (red = planning target volume, orange = simultaneous-integrated boost, pink = gross tumor volume).

We previously reported improved disease-free survival and PSA response from patients undergoing dose-escalated-SRT with SIB guided by PSMA-PET (^68^Ga-PSMA-11, ^18^F-rhPSMA-7, ^18^F-flotufolastat or ^18^F-PSMA-1007) compared with C-SRT^3^, without any significant increase in toxicity. Here we extend these findings in a cohort of patients treated based on PET imaging with recently approved radiopharmaceutical, ^18^F-flotufolastat, and the diastereoisomer mix from which it is derived, ^18^F-rhPSMA-7. The use of this class of radiopharmaceuticals has advantages based on the low level of urinary bladder activity allowing confident delineation of pelvic disease, particularly local recurrence for delivering SIB. Figure 6 shows the results of a ^18^F-flotufolastat PET/CT scan and corresponding contouring for SRT. The SRT was applied to the prostate bed with a SIB to the local recurrence located caudally to bladder.

Previous data from a prospective Phase 3 trial demonstrate the potential of ^18^F-flotufolastat to identify sites of recurrence missed by conventional imaging^25^. Our data suggest that this improved imaging may result in increased bFS among patients treated with ^18^F-rhPSMA-7/^18^F-flotufolastat-guided SRT compared with C-SRT. In all analyses conducted, the ^18^F-rhPSMA-7/^18^F-flotufolastat-guided SRT cohort showed an increased bFS, with the overall rate of bFS found to be higher at all evaluated timepoints in patients undergoing SRT guided by ^18^F-rhPSMA-7 or ^18^F-flotufolastat-guided than C-SRT. The most notable difference was among the subgroup who received SRT to the prostate bed only (*p* = 0.063). Moreover, and although fewer patients, we still observed an increased bFS among the subgroup who received the recently FDA-approved ^18^F-flotufolastat-guided SRT relative to patients receiving C-SRT.

However, since our data are retrospective and not powered to show superiority, the results should be interpreted with caution. The small sample size likely leads to larger 95% CI for the Cox regression analysis, and statistical significance was not reached. Nevertheless, the present data show potential for increased bFS in patients treated with ^18^F-rhPSMA-7/^18^F-flotufolastat-guided SRT cohort. With the possibility of generating SIBs to a local recurrence the radiation doses delivered to patients in our study were higher for patients receiving ^18^F-rhPSMA-7/^18^F-flotufolastat-guided SRT, which may suggest potential exists for greater improvements in bFS with increased doses to the macroscopic recurrence. A meta-analysis of over 10 000 patients by King et al. shows that SRT doses of > 70 Gy are associated with improved relapse-free survival^26^.

There are some limitations to the present analysis. First, as discussed above, our data are retrospective and not powered to show superiority, and thus future prospective studies are warranted to confirm these preliminary findings. However, the trend towards better outcomes for patients with ^18^F-rhPSMA-7/^18^F-flotufolastat-guided SRT is in line with prior data for other PET radiopharmaceuticals, and with data from our former study, which included limited cases of ^18^F-rhPSMA-7- and ^18^F-flotufolastat-guided SRT. It reported 86% of patients to show a PSA response of ≤ 0.2 ng/mL at last follow-up^3^. The EMPIRE-1 trial^27^ showed a significantly improved freedom from biochemical recurrence or persistence with radiotherapy guided by ^18^F-fluciclovine PET compared with those guided by conventional imaging (3-year failure-free survival of those undergoing ^18^F-fluciclovine PET was 76% compared with 63% for the patients undergoing conventional imaging (*p* = 0.003)). Moreover, an ongoing prospective phase III trial aims to evaluate ^68^Ga-PSMA-11-PET/CT-based SRT after radical prostatectomy and the results are eagerly anticipated (NCT03582774^28^). Second, our follow-up is relatively short, and a future analysis with longer follow-up is planned. As stated above, the follow-up is significantly longer in the C-SRT group than for patients with ^18^F-rhPSMA-7/^18^F-flotufolastat-guided SRT. However, this cannot be avoided since ^18^F-rhPSMA-7 and ^18^F-flotufolastat are newly developed tracers. Third, there is a difference in group size. This mainly results from not including patients with PSA recurrences below 0.2 ng/ml. However, this is necessary to enable comparability between the PET and non-PET groups. Median PSA value is significantly different between the two groups which can be explained by differences in the detection rates of PSMA imaging based on PSA values^15^. Fourth, in this analysis, we only report bFS since PSA follow-up is the standard aftercare strategy for patients after SRT. Further endpoints including imaging are planned to be part of a further multi-centric evaluation. However, they are prone to heterogeneity given the lack of standardized intervals for post-treatment imaging in early recurrent prostate cancer. Finally, although the cohorts are well balanced for most factors, the retrospective cohort design of our study is a limitation, although steps such as selection of patients with PSA levels ≥ 0.2 ng/mL, and the adjustment of the Cox regression analysis for the use of additive ADT, were taken to mitigate the impact of this. To further evaluate the hypotheses generated by this work, we plan a multi-national, multi-centric analysis. Nevertheless, we strongly believe that the presented real-world data can give a first outlook impression on outcome specifically using ^18^F-rhPSMA-7 or ^18^F-flotufolastat PET-guided RT.

## Conclusion

^18^F-Flotufolastat and ^18^F-rhPSMA-7 PET-guided SRT result in favorable disease outcomes in patients with biochemical recurrence of prostate cancer after radical prostatectomy. In our preliminary analysis, statistical significance was not reached, most likely due to the limited sample size. However, these data illustrate the potential for ^18^F-flotufolastat PET-guided SRT to be considered as part of personalized radiotherapeutic management of patients with PSMA-PET-positive local pelvic relapse after radical prostatectomy.

## Data Availability

Data are available from the corresponding author on reasonable request.
